# Manejo Invasivo versus Conservador de Pacientes com IAMSSST com Idade ≥ 75 Anos: Comentários

**DOI:** 10.36660/abc.20230364

**Published:** 2024-01-19

**Authors:** Sérgio Renato da Rosa Decker, Luiza Duarte Pittol, Regis Goulart Rosa, Marciane Maria Rover

**Affiliations:** 1 Hospital Moinhos de Vento Porto Alegre RS Brasil Serviço de Medicina Interna – Hospital Moinhos de Vento, Porto Alegre, RS – Brasil

**Keywords:** Infarto Agudo do Miocárdio sem Supradesnivelamento do Segmento ST, Idoso, Troponina, Isquemia, Mortalidade, Infarto do Miocárdio/prevenção e controle, Revascularização Miocárdica/cirurgia

Ao Editor,

Na edição dos Arquivos Brasileiros de Cardiologia, Hu M and Yang Y,^1^avaliaram o benefício de manejo invasivo precoce para pacientes idosos com infarto do miocárdio sem supradesnivelamento do segmento ST (IAMSSST), concluindo que essa estratégia produz resultados positivos na redução do infarto do miocárdio, eventos cardíacos adversos maiores e revascularização urgente. No entanto, existem algumas questões em relação à alegação de superioridade e à relevância dos desfechos escolhidos para essa população específica. Em primeiro lugar, esses desfechos (provocados pela isquemia) são, na sua maioria, desfechos substitutos de avaliação baseados nos níveis de troponina. Para estabelecer a substituição de um desfecho por outro, vários fatores precisam ser considerados, incluindo plausibilidade biológica, associações em estudos observacionais e, mais importante, prova de conceito por meio de ensaios onde a melhoria do desfecho substituto também melhora os resultados clínicos.^2^ Nesse sentido, as evidências mostram que a associação entre infarto do miocárdio não fatal e mortalidade cardiovascular ou por todas as causas atende aos dois primeiros níveis; porém, uma metanálise de ensaios clínicos randomizados não estabeleceu o infarto do miocárdio não fatal como um substituto para mortalidade cardiovascular ou por todas as causas.^3^ Na verdade, essa é uma relação reprodutível em desfechos de curto e longo prazo; a metanálise de FRISC-II, ICTUS e RITA-3 mostrou que, após cinco anos, a estratégia invasiva precoce para pacientes com IAMSSST não resultou em redução da mortalidade, apesar dos benefícios observados nos desfechos provocados pela isquemia.^4^

Em segundo lugar, esses desfechos substitutos utilizados podem ser suscetíveis a um viés de profecia autorrealizável. Em outras palavras, a previsão de que os pacientes submetidos à revascularização precoce não necessitarão de novas intervenções em um futuro próximo torna-se uma previsão cumprida; a revascularização não planejada e provocada pela isquemia é tanto a intervenção quanto o desfecho. Apesar disso, esses desfechos ainda podem ser justificados como substitutos para a qualidade de vida prejudicada e para o aumento da utilização de recursos de saúde, e ainda são importantes para intervenções preventivas primárias. No entanto, considerando pacientes com fragilidade no contexto do Sistema Único de Saúde (SUS), onde pacientes frequentemente enfrentam longos tempos de espera para cateterismo após IAMSSST, com aumento do tempo de internação hospitalar, a intervenção precoce padrão poderia piorar sua qualidade de vida e agregar danos como imobilidade relacionada à hospitalização. Dessa maneira, os desfechos relatados pelos pacientes, como angina ou qualidade de vida, por meio de questionários bem validados, podem servir como desfechos mais adequados para pesquisas nessa população e nesse contexto específicos. Infelizmente, esses desfechos centrados no paciente têm sido subnotificados nos estudos.^5^ Portanto, podemos interpretar os resultados de forma mais conservadora em relação à suposição de superioridade, particularmente nos casos de pacientes idosos que apresentam angina bem controlada e que estão em países de baixa a média renda com recursos limitados.
