# Supplementary material for: Manejo Invasivo versus Conservador de Pacientes com IAMSSST com Idade ≥ 75 Anos: Comentários
Source: Arq Bras Cardiol. 2024 Jan 19;120(12):e20230364. [Article in Portuguese] doi: 10.36660/abc.20230364 (PMC11021121; doi:10.36660/abc.20230364)
Supplement: Translation

# Translation: Invasive Versus Conservative Management of NSTEMI Patients Aged ≥ 75 Years: Commentary

**Authors:** Sérgio Renato da Rosa Decker, Luiza Duarte Pittol, Regis Goulart Rosa, Marciane Maria Rover

**Affiliations:** 1 Brazil Serviço de Medicina Interna – Hospital Moinhos de Vento, Porto Alegre, RS – Brazil

**Keywords:** Non-ST Elevated Myocardial Infarction, Aged, Troponin, Ischemia, Mortality, Myocardial Infarction/prevention and control, Myocardial Revascularization/surgery

Dear Editor,

In the edition of *Arquivos Brasileiros de Cardiologia*, Mengjin Hu et al.^1^ evaluated the benefit of early invasive strategy for elderly patients with non-ST-elevation myocardial infarction (NSTEMI), concluding that this strategy yields positive results in reducing myocardial infarction, major adverse cardiac events, and urgent revascularization outcomes. However, we have concerns regarding the authors’ claim of superiority and the relevance of the chosen outcomes for this specific population. Firstly, these outcomes (ischemia-driven outcomes) are mostly surrogate endpoints based on troponin levels. To establish the surrogacy of one outcome to another, several factors need to be considered, including biological plausibility, associations in observational studies, and, most importantly, proof of concept through trials where improving the surrogate endpoint also enhances clinical outcomes.^2^ In this sense, evidence shows that the association between nonfatal myocardial infarction and all-cause or cardiovascular mortality meets the first two levels; however, a meta-analysis of randomized clinical trials did not establish nonfatal myocardial infarction as a surrogate for all-cause or cardiovascular mortality.^3^ Indeed, this is a reproducible relationship in short and long term outcomes; the metanalysis of FRISC-II, ICTUS, and RITA-3 showed that, after five years, early invasive strategy for patients with NSTEMI did not result in reduced mortality despite the benefits seen in ischemia-driven outcomes.^4^

Secondly, these surrogate outcomes used may be susceptible to a self-fulfilling prophecy bias. In other words, the prediction that individuals who undergo early revascularization will not require further interventions in the near future becomes a fulfilled prediction; unplanned ischemia-driven revascularization is both the intervention and the outcome. Despite this, these endpoints may still be justified as surrogates for impaired quality of life and increased use of healthcare resources, and they are still important to primary preventive interventions. Nevertheless, considering frail patients in the context of the Brazilian Unified Health Systems (SUS), where patients often experience long waiting times for catheterization following NSTEMI, with increased length of hospital stay, the standard early intervention could worsen their quality of life and aggregate harms such as immobility related to hospitalization. Thus, patient-reported outcomes, such as angina or quality of life, according to well-validated questionnaires, might serve as more appropriate outcomes to research in this particular population and context. Unfortunately, these patient-centered outcomes have been underreported in studies.^5^ Therefore, we might interpret the results more conservatively regarding the assumption of superiority, particularly in the cases of elderly patients who have well-controlled angina and are in low- to middle-income countries with limited resources.
